# Irritability in stroke: a protocol for a prospective study

**DOI:** 10.3389/fneur.2024.1452491

**Published:** 2024-12-09

**Authors:** Wai Kwong Tang, Edward Hui, Thomas Wai Hong Leung

**Affiliations:** ^1^Department of Psychiatry, The Chinese University of Hong Kong, Hong Kong, Hong Kong SAR, China; ^2^Department of Imaging and Interventional Radiology, The Chinese University of Hong Kong, Hong Kong, Hong Kong SAR, China; ^3^Department of Medicine and Therapeutics, The Chinese University of Hong Kong, Hong Kong, Hong Kong SAR, China

**Keywords:** stroke, irritability, MRI, prefrontal cortex, anterior cingulate cortex, anterior temporal lobe, insula, amygdala

## Abstract

**Background:**

Poststroke irritability (PSI) is common among stroke survivors and can lead to a poor quality of life, difficulties in social interactions, criticism from caregivers, and caregiver stress. The planned study will evaluate the clinical, neuropsychological, and magnetic resonance imaging (MRI) correlates of PSI in a cohort of stroke survivors. In addition, the study will examine the 15-month progression of PSI.

**Methods:**

This will be a prospective cohort study that will recruit 285 participants. Participants and their caregivers will undergo detailed assessments at a research clinic at 3, 9, and 15 months after stroke onset (T1/T2/T3). The irritability/lability subscale of the Chinese version of the Neuropsychiatric Inventory (CNPI) will be completed by caregivers. Potential covariates will also be measured. Patients will undergo MRI, including diffusion-weighted imaging, within 1 week of stroke onset. A stepwise logistic regression will be performed to evaluate the importance of lesions in the regions of interest (ROIs) along with other significant variables identified in univariate analyses. These analyses will be repeated for patients with and without PSI at T2 and T3. Repeated measures analysis of covariance (ANCOVA) will be used to assess changes in CNPI scores for the entire sample. In ANCOVA analyses, the frequency of infarcts in the ROIs will be treated as the predictor.

**Discussion:**

This will be the first MRI study on PSI in stroke survivors. The findings will provide insights into the association of the orbitofrontal cortex, anterior cingulate cortex, anterior temporal lobe, insula, amygdala, thalamus, and basal ganglia lesions with the risk of PSI.

## Background

Irritability traditionally has been defined as a temporary psychological state characterized by impatience, intolerance, and poorly controlled anger, incorporating elements of aggression and decreased impulse control (1, 2). More recently, irritability has been described as a “mood that predisposes individuals toward certain emotions (e.g., anger), cognitions (e.g., hostile appraisals), and actions (e.g., aggression)” that is “subjectively unpleasant and objectively characterized by expressions of negative emotion in interpersonal relationships” (2, 3). Irritability is subjectively unpleasant and can either be brief or prolonged. Irritability should be distinguished from long-term traits, which are characterized by stable personality styles. Patients with irritability are often difficult to interact with, display emotional lability, and have outbursts in response to minor provocations. This neuropsychiatric symptom is strongly associated with functional disability (4).

In the context of psychiatric illnesses, irritability is a transdiagnostic phenomenon that occurs across all age groups and often leads to significant distress and impairment. Irritability is especially prevalent in mood disorders, where it plays a central role in diagnosis. Despite its ubiquity, irritability remains poorly understood (5). As a multidimensional construct, irritability is strongly associated with other psychological difficulties, such as depression and anxiety (6). There is considerable overlap between irritability and these psychiatric disorders, especially depression and anxiety. Many patients with depression report irritability, with nearly half experiencing a high level of this condition (7). In addition, irritability is listed as a symptom or associated feature of several anxiety disorders in the Diagnostic and Statistical Manual of Mental Disorders, Fourth Edition (8). A study indicated that clinically significant irritability is common in patients with anxiety disorders, particularly generalized anxiety disorder, where it is a diagnostic criterion (9). However, irritability can also occur independently of depression or anxiety (1, 2). Neurobiologically, irritability is associated with activity in the amygdala, orbitofrontal cortices, and hypothalamus. However, the patterns of activity in these areas differ from findings in youth. From a neurochemical perspective, irritability in adults is associated with increased monoamine transmission, whereas in youth, it involves abnormal dopamine (a monoamine transmitter) levels and responses (5).

Irritability is a common phenomenon in various cerebral diseases, such as Huntington’s disease (3, 4), traumatic brain injury (10, 11), dementia (12, 13), Parkinson’s disease (14, 15), multiple sclerosis (16, 17), and stroke (18, 19). For instance, the prevalence of irritability in Huntington’s disease ranges from 38 to 73% (3, 18). Similarly, irritability is frequently observed in patients with head injury, with a prevalence ranging from 15 to 74% (10, 11) and is associated with poor functioning and greater impairment in activities of daily living (20, 21). In patients with dementia or Huntington’s disease, irritability is a major source of distress for caregivers (22), often contributing to social and family dysfunction (3). In addition, irritability is a leading cause of hospitalization and institutionalization (23, 24). Studies have also linked irritability to suicidal ideation (25), violence (3) in patients, and harmful behavior by caregivers (26).

Poststroke irritability (PSI) is highly prevalent among stroke survivors, often manifesting as impatience over minor inconveniences (e.g., waiting or delays), flashes of anger (62%), rapid mood changes, and quarrels (29%) (27). Studies have reported that 29 to 70% of stroke patients experience irritability within 1 month to 7 years after stroke (19, 28–31). The frequency of PSI, as defined by the Neuropsychiatric Inventory (32), ranges from 12 to 53% (18, 27, 33, 34). A local study of 77 Chinese stroke survivors found a PSI prevalence of 9% (35). Possible clinical correlates of PSI include younger age, aphasia, premorbid personality traits (e.g., high neuroticism and low agreeableness) (27, 33), depressive and anxiety symptoms, and cognitive deficits (36). Irritability is particularly common in patients with poststroke aphasia, as impaired functional communication can lead to frustration and decreased tolerance for trivial matters (19). In addition, irritability is a common symptom in dementia, affecting nearly 30% of patients (35), and is found in 13–45% of patients with mild cognitive impairment (37). Furthermore, irritability may reflect difficulties in coping with newly acquired stroke-related disabilities. Even after a mild stroke, survivors often struggle to cope with the uncertainty of their condition, which can contribute to irritability (38). Despite its prevalence, PSI is often undiagnosed and thus untreated in stroke survivors (17), leading to poor recovery (39) and quality of life (36), difficulties in social interactions (40), criticism from caregivers (41), and caregiver stress (42).

The course of PSI remains unclear. In a study including 128 stroke patients, irritability was observed in 40, 40, and 34% of patients at 1, 6, and 12 months after stroke, respectively (29). Another study involving 124 stroke survivors reported that PSI may peak at 1 year poststroke, indicating its potential chronicity (27). A pilot study of 11 stroke survivors with PSI reported a remission rate of 63% at the 1-year follow-up (34). In addition, in a 2-year longitudinal study, recovery rates for poststroke depression (PSD) were 75% for dysthymia, 100% for minor depression, and 75% for major depression. Recovery rates for poststroke anxiety were 83% for panic disorder, 60% for generalized anxiety disorder, and 50% for social phobia (43).

To date, no high-quality trials have been conducted on pharmacological or psychosocial treatments for irritability in stroke patients. Although carbamazepine may be effective in managing PSI (44), antidepressants have not shown efficacy (36). Studies on patients with Huntington’s disease have indicated that serotonin reuptake inhibitors, valproate, atypical antipsychotics, beta-blockers, and synthetic cannabinoids may be effective in treating apathy and aggression (3, 4, 45). Similarly, studies on patients with traumatic head injury have suggested that amantadine (37) and sertraline may be effective for treating irritability (46–48). Regarding nonpharmacological interventions, validation therapy, music therapy, aromatherapy, and cognitive-behavioral interventions may offer benefits (49, 50). A recent review of PSD treatments supported the efficacy and safety of selective serotonin reuptake inhibitors (SSRIs) and recommended nortriptyline for patients who do not respond to SSRIs. Cognitive behavioral therapy (CBT), virtual reality, and repetitive transcranial magnetic stimulation (rTMS) have also shown promise in treating PSD. CBT, a widely recognized psychotherapeutic intervention, helps individuals reshape negative thought patterns and develop effective coping strategies tailored to their unique poststroke challenges. rTMS, a noninvasive brain stimulation technique, demonstrated effectiveness in rebalancing neural activity and reducing depressive symptoms in stroke survivors (51). In addition, some clinical trials have suggested that acupuncture and reminiscence therapy are effective in treating poststroke anxiety (52, 53).

Starkstein and Robinson (54) indicated that PSI may result from damage to the orbito-temporal-limbic feedback loop, where the inhibitory control of the cortex over the amygdala is disrupted, reducing the ability to suppress instinctive emotional reactions. The key components of this brain circuit are the medial orbitofrontal cortex (OFC), amygdala, and connecting tracts (55). In Huntington’s disease, irritability is caused by disruptions in the emotional circuitry involving the medial OFC and amygdala (56). A functional magnetic resonance imaging (fMRI) study of the brain’s response to frustration revealed that exposure to frustrating stimuli leads to increased activation in various regions, including the amygdala and dorsomedial and ventromedial prefrontal cortices (57). Motivational deficits in PSD were associated with lesions in the OFC (58). The relationship between irritability and dysfunctions in the primary components of the fronto-amygdala circuit is discussed in the following paragraphs.

Irritability is commonly observed in patients with frontal lobe pathologies, such as frontotemporal dementia (13, 59). A single case report demonstrated a relationship between irritability and frontal lobe stroke (44). Furthermore, irritability is a common sequela following traumatic frontal lobe injury (60–63). Irritability has been associated with damage to the OFC (64–66). Moreover, in primary progressive aphasia, irritability was related to atrophy of the lateral OFC and anterior cingulate cortex (ACC) (67). An fMRI study on Huntington’s disease reported that irritability results from volume reduction and dysfunction in the medial OFC (56, 68). In addition, fMRI studies have shown that individuals with chronic irritability exhibited dysfunctions in the medial frontal gyrus (69), middle/superior frontal gyrus (70), inferior frontal gyrus (49), and ACC (69, 71). ACC lesions and decreased functional connectivity have also been associated with PSD (72, 73).

In addition to the frontal cortex, temporal lobe structures have been implicated in the pathophysiology of irritability. In patients with traumatic head injury, Gualtieri (65) observed an association of irritability with damage to the anterior temporal lobe (ATL). In individuals with chronic irritability, a reduction in gray matter volume was observed in the insula (49). Irritability in patients with Alzheimer’s disease (AD) has been associated with insula atrophy (74) and abnormal functional connectivity (75). Task-related fMRI studies of facial emotion processing in individuals with chronic irritability have shown dysfunctions in the superior temporal gyrus (76) and posterior insula (77).

The amygdala plays a central role in regulating emotions and is implicated in the pathophysiology of irritability in various clinical populations (78). In patients with dementia, atrophy of the amygdala was associated with the severity of irritability (79). The volume of the amygdala was significantly decreased in patients with dysphoric disorder associated with epilepsy, and this reduction was correlated with the level of irritability (80). An fMRI study in patients with AD found that amygdala dysfunction and hypersensitivity were correlated with the severity of irritability (81). In addition, several fMRI studies using facial emotion processing tasks have shown that individuals with chronic irritability exhibited dysfunction in the amygdala (70, 77, 82, 83). In poststroke patients, somatic symptoms associated with depressive states are related to amygdala lesions (58, 84).

Evidence suggests that decreased white matter integrity is associated with irritability in neuropsychiatric disorders (85). In a diffusion tensor imaging study of 45 individuals with mild cognitive impairment or AD, lower integrity of the anterior cingulum, as measured through fractional anisotropy, was related to a higher risk of irritability (86). In a study on depression, irritability was found to be associated with decreased integrity in the anterior corona radiata, inferior longitudinal fasciculus, and inferior fronto-occipital fasciculus (55). In addition, white matter structural and functional changes are also associated with PSD (87–89).

Irritability is a known feature of basal ganglia (BG) disorders (3, 4, 14, 90, 91). One possible explanation for irritability in BG disorders is the development of “rigidity” in thinking, where patients fixate on a particular desire or idea, leading to outbursts when their perceived needs are unmet (91). Individuals with chronic irritability have a decreased gray matter volume in the globus pallidus (49). A case report indicated an association of irritability with an infarction in the left subthalamic nucleus (92). Task-related fMRI studies have also revealed dysfunction in the BG in individuals with chronic irritability (49, 83, 93, 94). In addition, BG lesions are associated with poststroke depressive symptoms (95–97).

Many neuroimaging studies on poststroke anxiety and depression have been published (58, 89). However, few structural brain imaging studies have focused on irritability following stroke (28, 36). Single case reports have demonstrated an association of irritability with anterior (98), paramedian thalamic (99), and subthalamic (92) infarctions. In a study including 16 patients with malignant middle cerebral artery infarction (100), 53% showed an increase in irritability. Folstein et al. (28) reported that 70% of patients with right hemisphere stroke exhibited irritability, whereas no patients with left hemisphere stroke displayed irritability. Chan et al. (36) found that irritability in 92 stroke patients was related to lesions near the frontal pole. However, these studies have several limitations, including small sample sizes (28, 36, 100), the inclusion of participants with current psychiatric diagnoses (36), and a lack of detailed radiological examinations. Furthermore, the classification of infarct locations is often crude, such as hemispheric versus brainstem (36), left versus right (28), and anterior versus posterior (36).

## Aims and hypotheses to be tested

The primary objective of the proposed study is to evaluate the clinical and magnetic resonance imaging (MRI) correlates of PSI in a cohort of stroke survivors, an area that has not been explored in published studies. The secondary objective is to examine the 12-month progression of PSI.

### Hypotheses

The first hypothesis is that individuals with PSI have more infarcts in the regions of interest (ROIs) but not in the control region than those without PSI. The ROIs will be the OFC, ACC, ATL, insula, amygdala, thalamus, and BG. The occipital lobe will be included as the control region. The second hypothesis is that the number of infarcts in the ROIs is significantly and positively correlated with the severity of PSI. The third hypothesis is that 37% (34) of individuals with PSI at baseline continue to exhibit PSI 12 months after the initial assessment.

## Methods

### Participant recruitment

**Figure 1:**
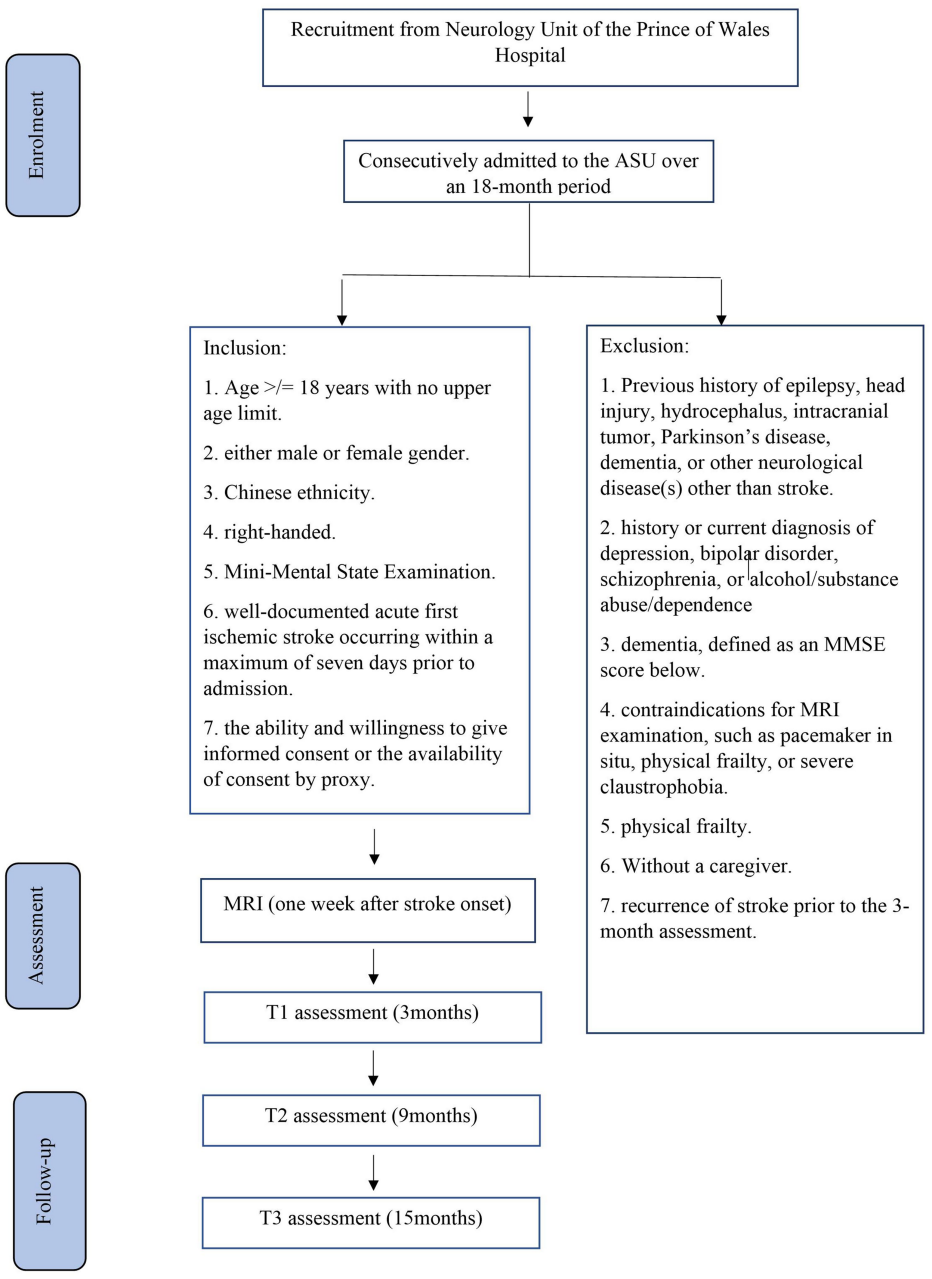
Details of recruitment.

The planned study is a prospective nested case–control study of stroke survivors. Details of the recruitment process are shown in Figure 1. Participants will be recruited from patients consecutively admitted with a first-ever stroke to the Acute Stroke Unit (ASU) at the Prince of Wales Hospital (PWH). The PWH is a general hospital serving a population of 800,000 in Hong Kong. The ASU treats approximately 93% of all patients with acute stroke admitted to the PWH, with the remaining 7% admitted to the neurosurgery unit. All patients with acute stroke (*n* = 1,000) who are consecutively admitted to the ASU over a 24-month period will be invited to participate. A trained research assistant (RA) will visit the ASU daily to identify eligible patients and obtain written consent. Approximately 80% of these 1,000 patients (*n* = 1,000 × 80% = 800) are expected to have ischemic stroke. MRI examination will be contraindicated in 10% of these patients, leaving 720 potential participants (800 × 90%). Based on our previous findings (101), the mortality rate at 3 months poststroke is approximately 12%, reducing the number of potential participants to 634 [720 × (100% − 12%)]. Assuming a 25% dropout rate and that approximately 60% of the survivors will meet the inclusion criteria (101), we will recruit approximately 285 participants [634 × (100% − 25%) × 60%]. Follow-up assessments at 6 and 12 months will be conducted for all 285 participants.

### Eligibility criteria

#### Inclusion and exclusion criteria

The inclusion criteria will be as follows: (1) age ≥ 18 years with no upper limit; (2) either male or female sex; (3) Chinese ethnicity; (4) right-handed; (5) a clinical diagnosis of acute first ischemic stroke occurring within a maximum of 7 days prior to admission, as recorded in the medical notes; and (6) ability and willingness to provide informed consent or availability of consent by proxy.

The exclusion criteria will be as follows: (1) previous history of epilepsy, head injury, hydrocephalus, intracranial tumor, Parkinson’s disease, dementia, or any other neurological diseases other than stroke; (2) history or current diagnosis of depression, bipolar disorder, schizophrenia, or alcohol/substance abuse or dependence; (3) dementia; (4) contraindications for MRI examination, such as the presence of a pacemaker, physical frailty that prevents attendance at the research clinic, or severe claustrophobia; (5) physical frailty; (6) lack of a caregiver; and (7) recurrence of stroke prior to the 3-month assessment.

### Data collection

The data collection schedule is presented in Table 1. Written consent will be obtained from all patients. The number of exclusions and reasons for exclusion will be recorded. The following demographic, psychosocial, and medical data will be collected from all participants: age, sex, education level, and date of stroke onset. Clinical data, including information on neurological impairments such as aphasia and dysarthria, will be measured using the National Institute of Health Stroke Scale (NIHSS) (102) and extracted from the Stroke Registry, which is maintained by a full-time trained research nurse.

**Table 1:** Data collection schedule.

Study period	T0	T1	T2	T3
Months	0	3	9	15
Visits	0	1	2	3
MRI	X			
Review of inclusion/exclusion criteria	X			
Informed consent	X			
IDA, BI, MMSE, BDI, HADSA		X	X	X

### Assessment of PSI

#### Assessment of irritability

Three months after the onset of the index stroke (T1), patients and their caregivers will undergo assessments at a research clinic. The timing of the assessments will be consistent with other studies on PSI (31, 33).

A trained RA will conduct a clinical interview at the research clinic. PSI will be evaluated using the irritability/lability subscale of the Chinese version of the Neuropsychiatric Inventory (CNPI). The CNPI is based on a structured interview with the caregiver, during which a screening question is asked to determine the presence or absence of irritability over the past month (32, 103). The behavior must reflect a change from the patient’s pre-stroke condition. If the response is positive, the behavior will be further explored with sub-questions. Frequency is rated from 1 to 4, and severity is scored from 1 to 3. The product of severity and frequency is calculated to quantify the severity of PSI. The Neuropsychiatric Inventory is the most commonly used tool for assessing irritability in stroke (18, 27, 33, 34, 104).

Physical functioning, depressive and anxiety symptoms, aphasia, and cognitive functioning will be assessed using the Barthel Index (BI) (105), the Beck Depression Inventory (BDI) (106), the anxiety subscale of the Hospital Anxiety Depression Scale (HADSA) (107), the Language Screening Test (LAST) (108), and the Montreal Cognitive Assessment (MoCA) (109), respectively. For participants with substantial aphasia, proxy ratings will be obtained from caregivers. Pre-stroke personality will be assessed using the Chinese version of the NEO-Five-Factor Inventory, which will be completed by the caregiver (110, 111).

Follow-up assessments of irritability will be conducted for all participants at 9 months (T2) and 15 months (T3) poststroke, corresponding to 6 and 12 months after the initial assessment. Any new episode of depression or anxiety disorder, as well as subsequent pharmacological treatments, will be recorded. The assessments (BI, MoCA, BDI, and HADSA) will be repeated during follow-up (Table 1).

### MRI examination and analysis

Stroke patients will undergo MRI within 1 week of stroke onset. All scans will be performed using a 3 T scanner (Philips Achieve 3.0 T, X Series, Quasar Dual MRI System) with standardized sequences, including diffusion-weighted imaging (DWI); three-dimensional T1-weighted, T2-weighted, fluid-attenuated inversion recovery (FLAIR); and susceptibility-weighted imaging (SWI). An experienced neuroradiologist blinded to the participants’ psychiatric diagnoses and PSI status will assess the MRI images. Acute infarcts will be identified as hyperintense lesions on DWI with corresponding hypointensity on the apparent diffusion coefficient map. White matter hyperintensities (WMHs) will be defined as ill-defined hyperintensities 5 mm on FLAIR, which are isointense with a normal brain parenchyma on T1-weighted images. Lesions that exhibit signal characteristics of the cerebrospinal fluid on T1-weighted images and measure more than 3 mm in diameter, as well as wedge-shaped cortico-subcortical lesions, will be classified as old/lacunar infarcts. Microbleeds will be defined as dot-like hypointense regions on SWI, and the total number of microbleeds will be determined. The number of microbleeds in the BG and thalamus will be noted separately. All raw data will be transferred to the PALS system (Carestream Solutions).

#### MRI preprocessing

Preprocessing steps will include nonuniformity correction (112), spatial standardization, and brain extraction (removal of the skull). To ensure that brain structure volumes are comparable among participants, the MRI data of each participant will be transformed from its original space to a common stereotactic space using multiscale affine registration (113). Brain regions will be automatically segmented from the MRI data by using the brain extraction tool (114).

#### Brain segmentation

The brain tissue will be classified into gray matter, white matter, and cerebrospinal fluid (115). Whole-brain segmentation will be conducted using an atlas-based approach (116), which automatically adjusts the existing atlas intensity model to new data. The ROIs and other brain regions will be segmented, and their volumes will be quantified using the Taahirah brain atlas (107) and demon registration (117).

#### Infarct segmentation and quantification

Infarcts will be delineated semi-automatically as high-intensity regions on DWI, whereas WMHs will be identified as high-density regions on FLAIR (and isointense on T1-weighted images) using ITK-SNAP software. The segmented infarct and WMH regions will be combined with the ROIs and other brain-region masks generated in the previous step. The infarct and WMH pixels within the ROIs and other brain regions will then be calculated.

### Sample size estimation

A total of 287 patients will be recruited, with the expectation that 22% will present with PSI, resulting in 63 patients with PSI (18, 27, 33, 34, 104). If no significant correlations between irritability and lesion location are found in this sample, it is unlikely that clinically meaningful effects of lesions would be detected in a larger sample. Because no published data exist on the specific locations of infarcts in PSI, our estimates were based on figures reported for other poststroke neurobehavioral disorders. Tang et al. (118) reported that frontal infarcts were present in 15 and 5% of patients with and without poststroke emotional incontinence (PSEI), respectively. In another study on poststroke anxiety (PSAn), 21.4% of patients with PSAn had frontal infarcts, compared with only 8.6% of patients without PSAn (119). Similar to PSI, frontal lobe dysfunction is believed to be involved in PSEI and PSAn. A sample size of 285 will provide 80–86% power to identify frontal infarcts as a predictor of irritability in stroke based on a one-degree-of-freedom chi square test (120).

### Statistical analysis

All variables will be tested for normality using the Kolmogorov–Smirnov test with a significance threshold of *p* < 0.05. Demographic, clinical, and MRI variables (age, sex, NIHSS, BI, HADSA, BDI, MoCA, and NEO-Five-Factor Inventory scores; infarcts in ROIs; microbleeds; and WMH volumes) will be compared between groups using the chi-square test, Student’s t test, or Mann–Whitney U test, as appropriate. A stepwise logistic regression will be performed to evaluate the importance of lesions in the ROIs, along with other significant variables identified in the univariate analyses. These analyses will be repeated for patients with and without PSI at T2 and T3.

Repeated measures analysis of covariance (ANCOVA) will be used to assess changes in the CNPI scores for the entire sample. In the ANCOVA analysis, the frequency of infarcts in the ROIs will be treated as the predictor, and the statistical model will be adjusted for age, aphasia (LAST), personality traits (NEO-Five-Factor Inventory scores), cognitive function (MoCA score), depressive and anxiety symptoms (BDI and HADSA scores), new episodes of anxiety disorder, and subsequent pharmacological treatments. At each time point, the CNPI scores will be analyzed using linear regression while controlling for the same covariates as in the ANCOVA. Multiple testing will be controlled using false discovery rate correction. The level of significance will be set at 0.05.

## Discussion

We aim to include a homogenous patient population by narrowing the criteria for age, ethnicity, handedness, and PSI duration. Patients with other causes of PSI, such as psychiatric or neurological disorders, will be excluded. This will be the first longitudinal study to examine the role of the OFC, ACC, ATL, insula, amygdala, thalamus, and BG in a large sample of consecutively admitted stroke survivors with PSI. The results will provide insights into the association between these brain regions and PSI. The findings will be applicable to a broad population of neurological patients at risk of irritability and stimulate further research in this field.
